# Enhanced Activity of Au/NiO Nanohybrids for the Reductive Amination of Benzyl Alcohol

**DOI:** 10.3390/ma10121435

**Published:** 2017-12-16

**Authors:** Carine E. Chan-Thaw, Lidia E. Chinchilla, Felipe Juan Sanchez Trujillo, Nikolaos Dimitratos, Gianluigi A. Botton, Laura Prati, Alberto Villa

**Affiliations:** 1 Department of Chemistry, Università degli Studi di Milano via Golgi 19, 20133 Milan, Italy; Carine.Chanthaw@unimi.it (C.E.C.-T.); Laura.Prati@unimi.it (L.P.); 2 Canadian Centre of Electron Microscopy and Department of Materials Science and Engineering, McMaster University, 1280 Main Street West, Hamilton, ON L8S 4M1, Canada; lidia.chinchilla@uca.es (L.E.C.); gbotton@mcmaster.ca (G.A.B.); 3 Cardiff Catalysis Institute, School of Chemistry, Cardiff University, Main Building, Park Place, Cardiff CF103AT, UK; SanchezF@cardiff.ac.uk

**Keywords:** gold, NiO nanohybrids, reductive amination

## Abstract

Gold nanoparticles were prepared by sol immobilization (Au_SI_) or deposition precipitation (Au_DP_), then deposited on NiO and commercial TiO_2_ (P25). The Au/NiO catalysts showed higher activity and yield to the secondary amine, compared to Au/TiO_2_ catalysts, when tested for the reductive amination of benzyl alcohol with isopropylamine. We attribute this result to a synergistic effect between Au and NiO. Moreover, as a result of the protective effect of the polyvinyl alcohol used in the sol immobilization synthesis, the gold nanoparticles on NiO demonstrate an increased resistance to structural changes during the reaction. This effect results in enhanced catalytic efficiency in terms of activity, and better stability against deactivation.

## 1. Introduction

**Scheme 1:**
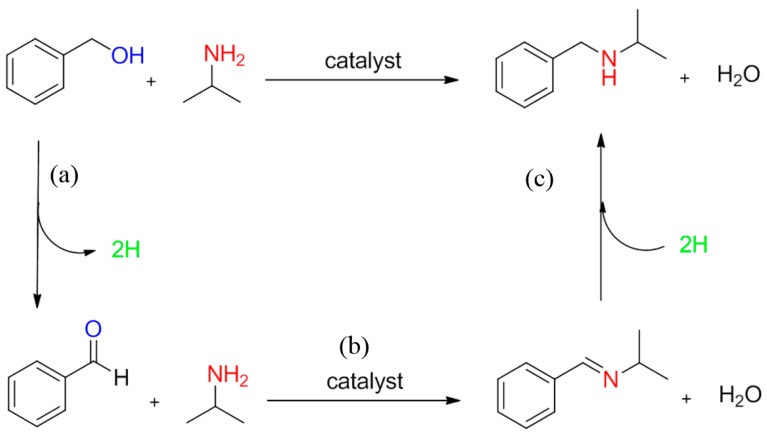
Accepted mechanism of *N*-alkylation of isopropylamine with benzyl alcohol.

Secondary amines are important intermediates in the formation of fine valuable chemicals produced by organic synthesis. These amines are important pharmacophores, dyes, agrochemicals, surfactants, fine chemicals, and functionalized chemicals [1,2]. The most common and straightforward method to form secondary amines is the *N*-alkylation of primary amines with alkyl halides in the presence of a stoichiometric amount of base. Over-alkylation occurs at the same time, and leads to the significant production of by-products. A mixture of primary, secondary, and tertiary amines, as well as quaternary ammonium salts, results from this method. Moreover, the toxicity of many alkyl halides and related alkylating agents has been established [3]. Secondary amines can also be obtained in the presence of a reducing agent or hydrogen gas through the reductive amination of carbonyl compounds (such as aldehyde or ketones) with primary amines via the formation of an imine [3]. However, reductive amination of alcohols is preferred to carbonyl groups, as the latter are derived from the oxidation of alcohols. An alternative approach, involves the reductive amination of primary alcohols to produce secondary amines [4], which is an environmentally friendly method with only water as a by-product. This one-pot process can be described in three distinct steps: (a) the dehydrogenation of the alcohol to the corresponding aldehyde; (b) the dehydrated condensation of the aldehyde with the primary amine to the imine through a hemiaminal intermediate; and finally (c) hydrogenation of the imine to form the secondary amine (Scheme 1, case of isopropylamine with benzyl alcohol). Ir [5] or Ru [6] based homogeneous systems have been reported, with the main challenge of recovery and reuse of the catalyst. Heterogeneous metal catalysts, contrary to the homogeneous ones, have a better tolerance to high temperature and pressure, while some have a lower cost, and are easily removed from the reaction mixture for reuse.

In the last few years, heterogeneous metal catalysts, such as Ru [7], Pd [8,9,10] and Cu [11], were reported as being active in the *N*-alkylation of amines with alcohol. However, high catalytic activity and selectivity require an excess of amines or alcohols in the presence of acidic or basic additives. Heterogeneous Ni catalysts were also reported as active for the direct synthesis of primary amines from alcohols and ammonia [12], as well as for the *N*-alkylation of amines with alcohols [13].

Gold based catalysts are known to be highly active for the selective oxidation of alcohols [14], for the hydrogenation of aldehydes, ketones, and imines [15], and to perform the oxidant-free dehydrogenation of alcohols under mild conditions [16]. As a result, heterogeneous gold catalysts have attracted interest for the direct alkylation of amines by alcohols [17,18,19,20,21]. Materials based on Au NPs were demonstrated to perform the *N*-alkylation of amines with alcohols by hydrogen transfer under inert atmosphere in one pot [17,18,21,22]. There is no need to provide external hydrogen, as the hydrogen produced by the dehydrogenation of alcohol to aldehyde can be used later for the reduction of the imine to the secondary amine (Steps a and c in Scheme 1). Haruta et al. demonstrated that gold clusters deposited by a solid grinding method on porous coordinated polymers showed a high catalytic performance in the one-pot synthesis of secondary amines [17]. He et al. [21] reported the best activity and selectivity to the target molecule, namely the secondary amine, with Au clusters supported on TiO_2_. Later, Haruta and coworkers demonstrated that Au/ZrO_2_ is a promising catalyst in the *N*-alkylation of aniline with benzyl alcohol to secondary amine [18]. In both cases, they were using equimolar amounts of substrates without additives under N_2_ at atmospheric pressure.

We have recently demonstrated that gold nanoparticles deposited on NiO showed better activity compared to Au/TiO_2_ in the benzyl alcohol oxidative dehydrogenation, due to a cooperative effect between Au nanoparticles and nanosized NiO [23,24]. As the dehydrogenation of benzyl alcohol is the first step of the one-pot *N*-alkylation of primary amines with benzyl alcohol, in this work, the same supports have been investigated, and their activity compared.

## 2. Results

**Figure 1:**
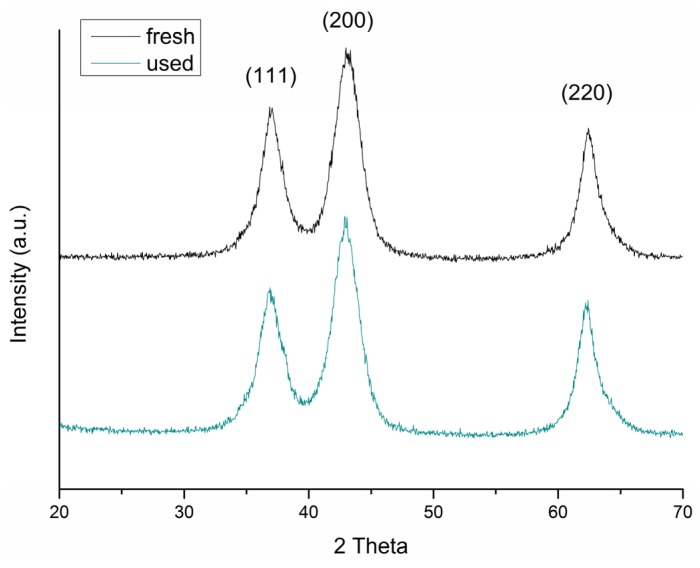
XRD of the fresh and used Au_SI_/NiO catalyst after six cycles.

Nickel oxide was prepared following a procedure reported elsewhere [23]. X-ray diffraction confirmed the presence of NiO with average crystallite size of 5.5 nm, calculated from measured values for the spacing of the (111) planes using Scherrer equation (Figure 1). Following methods previously reported, 1% Au on NiO and TiO_2_ catalysts were prepared by deposition precipitation with urea (DP), and by sol immobilization using polyvinyl alcohol (PVA) as stabilizer (SI) [14,25]. We have chosen these preparation methods to investigate the performance of Au nanoparticles in presence (PVA) and in absence (DP) of a protective agent. Indeed, we have shown that the presence of a protective agent can modify the activity and the selectivity in alcohol oxidation [25]. The metal content analyzed by atomic absorption spectroscopy (AAS) was approximately the nominal value of 1 wt %, except for Au_DP_/NiO, where the measured loading was 0.7%. Detailed characterization of the Au nanoparticles on TiO_2_ was reported elsewhere [25], and showed an average particle size of 3.8 and 3.5 nm for 1% Au_DP_/TiO_2_ and 1% Au_SI_/TiO_2_, respectively (Table 1).

**Figure 2:**
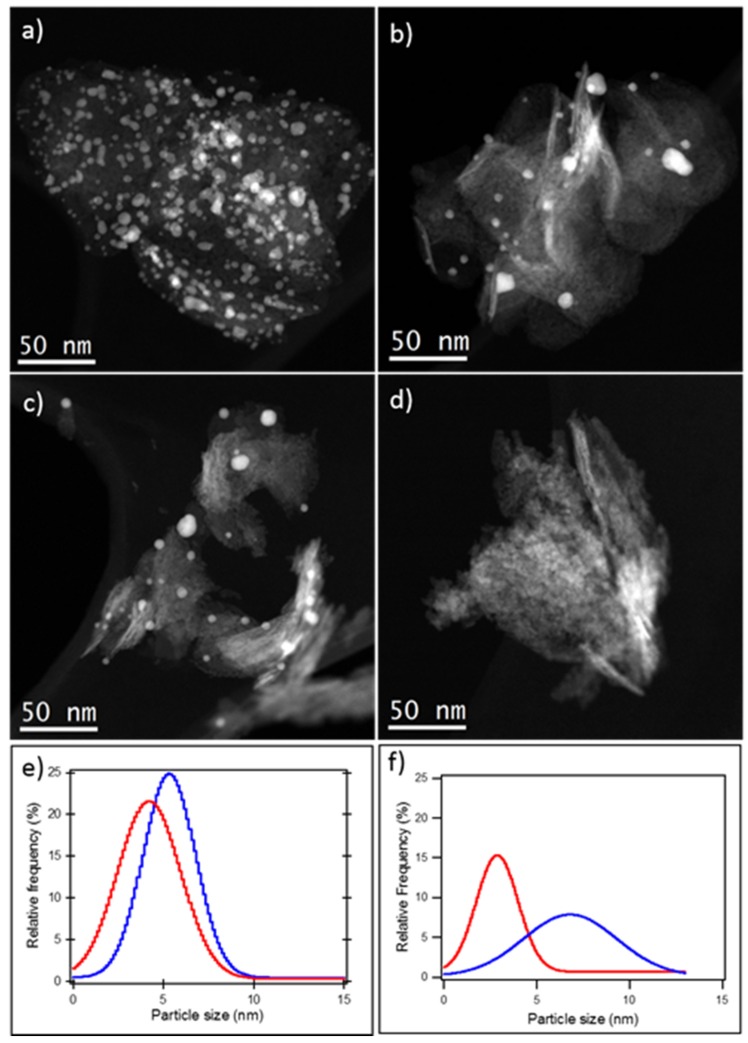
Low magnification HAADF-STEM images of fresh (**a**) Au_SI_/NiO and (**b**) Au_DP_/NiO versus (**c**) Au_SI_/NiO and (**d**) Au_DP_/NiO, after reaction. Comparison of the Gaussian fit to the respective particle sizes determined from TEM images of the fresh catalyst (red), and after reaction (blue); Au_SI_/NiO in (**e**) and Au_DP_/NiO in (**f**).

As the intensity of HAADF-STEM images is roughly proportional to the atomic number of the elements present in the sample, this technique is suitable to distinguish between the particles containing the heavier element, gold, and the lighter regions containing the nickel-based support. The HAADF-STEM image of the 1% Au_SI_/NiO catalyst shown in Figure 2a clearly illustrates well-distributed gold nanoparticles supported on NiO. A corresponding statistical analysis of 350 particle diameters in several images reveals that gold particles ranging from 1 to 8 nm were effectively deposited onto the NiO support using the sol-immobilization technique, with an average particle size of 4.8 (Table 1). Preparing the gold catalysts by the deposition precipitation technique results in a similar gold particle distribution, but smaller particle size (3.4 nm) (Table 1). Table 1 gathers the averaged diameters of the particle size distribution (PSD) and the resulting standard deviation determined by TEM of all catalyst prepared for this investigation. Figure 2 clearly shows also the presence of few large Au aggregates in both Au_SI_/NiO and Au_DP_/NiO. For the particle calculation, the single particles, and not the entire aggregates, have been considered.

The catalysts were then tested for use in the amination of benzyl alcohol with isopropylamine, to form *N*-isopropylbenzylamine, an intermediate in the pharmaceutical industry [1,3] (Scheme 1). To the best of our knowledge, this is the first example of production of *N*-isopropylbenzylamine via reductive amination using gold catalysts. To evaluate any possible contribution of the support, bare TiO_2_ and NiO were also tested (Table 2). TiO_2_ was inactive, however, NiO showed a conversion of 29% with a 95% selectivity to the imine (Table 2). NiO is known to be active for the dehydrogenation of benzyl alcohol, [23] however, under these reaction conditions, NiO is not able to perform the step of hydrogenation of the intermediate imine to amine. In the case of Au supported on TiO_2_, the benzyl alcohol conversion after 24 h of reaction time showed similar levels of conversion for both preparation methods (38% and 32% for 1% Au_DP_/TiO_2_ and 1% Au_SI_/TiO_2_, respectively; Table 2). Au_SI_/TiO_2_ (1%) showed a relatively lower activity in the hydrogenation step and lower selectivity to *N*-isopropylbenzylamine at 33%, compared to 1% Au_DP_/TiO_2_.

In comparison, Au/NiO catalysts were more active than the corresponding Au/TiO_2_ ones. This result can be due to a synergistic effect of Au and NiO, both active sites for the dehydrogenation step. The catalyst prepared by sol immobilization showed a better activity than the one prepared by deposition precipitation, with a conversion of 58% and >99% after 24 h for 0.7% Au_DP_/NiO and 1% Au_SI_/NiO, respectively.

**Figure 3:**
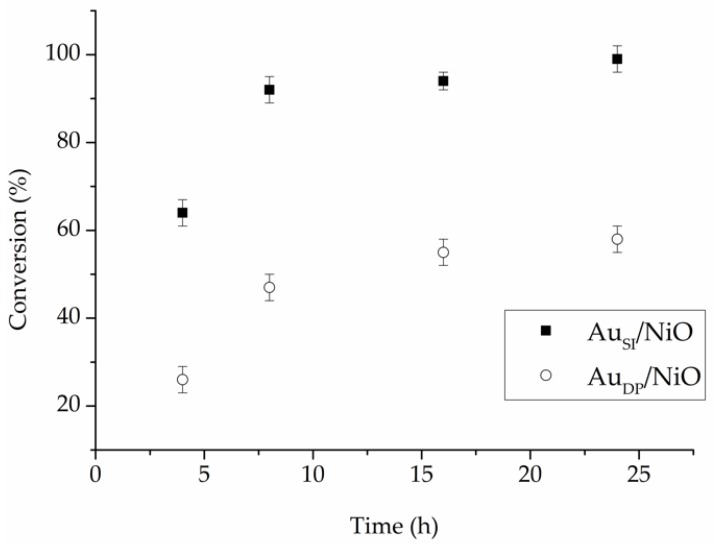
Reaction profile of Au_SI_/NiO and Au_DP_/NiO. Reaction conditions: isopropylamine (7 mmol), benzyl alcohol (7 mmol), total volume in xylene (30 mL), BA/Au: 250 mol %, 150 °C, 5 atm, N_2_, 1200 rpm.

Plotting the conversion versus reaction time profile showed the 1% Au_SI_/NiO reached 92% conversion after 8 h (Figure 3). Moreover, a significant deactivation was observed in the case of 0.7% Au_DP_/NiO after 16 h of reaction (Figure 3).

In order to correlate the decreased catalytic performance with structural changes of the nanoparticles, the catalyst after reaction was characterized by (S)TEM. A representative HAADF-STEM image of Au_DP_/NiO after reaction in Figure 2d shows that the loss of catalytic activity may be due leaching of Au into the solution during the reaction. AAS experiments confirmed a leaching of 60% of Au nanoparticles during the reaction. Particles may be lost due to particle detachment and/or gold leaching. In addition, gold agglomeration occurred, therefore, the average particle size increased by 115%, as is shown in Table 1. Consequently, the decreased number of particles and particle agglomeration leads to a lower number of exposed active sites, resulting in a lower activity and in a significant deactivation of the system.

**Figure 4:**
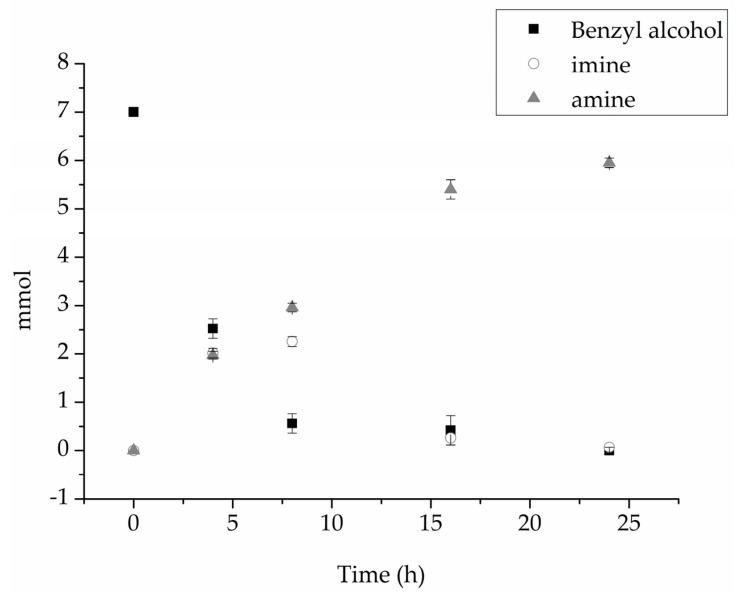
Product distribution for Au_SI_/NiO. Reaction conditions: isopropylamine (7 mmol), benzyl alcohol (7 mmol), total volume in xylene (30 mL), BA/Au: 250 mol %, 150 °C, 5 atm, N_2_, 1200 rpm.

For the Au/NiO catalyst, after 24 h, *N*-isopropylbenzylamine was the main product in both cases. In particular, Au_SI_/NiO showed with a selectivity of 85% at 99% conversion (Table 2). The product distribution for Au_SI_/NiO revealed an initial production of the same amount of imine and amine after 4 and 8 h of reaction, whereupon all the imine is reduced to the corresponding amine (Figure 4). The presence of a protective agent seems to be essential to stabilize Au nanoparticles during the reaction, and to prevent leaching, and therefore, deactivation of the catalytic system.

**Scheme 2:**
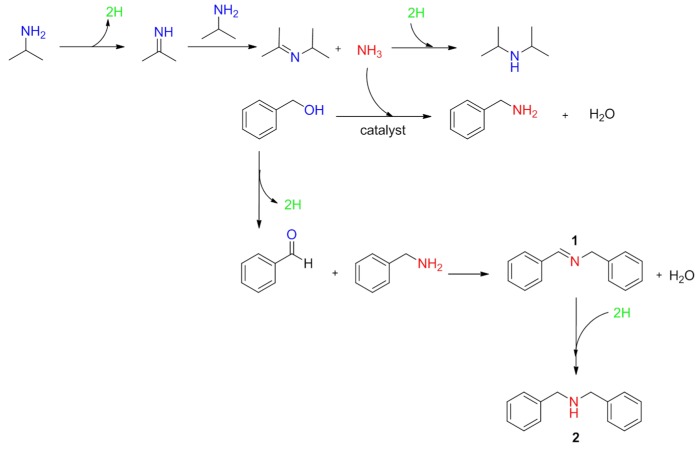
Possible parallel reactions during reductive amination of benzyl alcohol with isopropylamine.

In the case of NiO and Au_SI_/NiO, benzenemethanamine *N*-(phenylmethylene) and dibenzylamine were formed as the main side products (1) and (2), respectively (Table 1, Scheme 2) as confirmed by GC-MS. The proposed reaction pathway to form these side products is depicted in Scheme 2. During the first step, a self-condensation of the isopropylamine occurred to form the secondary symmetric di-isopropylamine and NH_3_. The latter species reacts directly with benzyl alcohol to form benzylamine. Benzylamine, together with benzaldehyde, which is obtained by the dehydrogenation of benzyl alcohol (Scheme 1a), are converted to 1 (Scheme 2). According to the hydrogen transfer described in Scheme 1, the imine (1) is hydrogenated to the secondary amine (2), namely dibenzylamine.

To corroborate the proposed mechanism, we performed a reaction using benzyl alcohol solution mixed with gaseous NH_3_, obtaining benzenemethanamine *N*-(phenylmethylene) and dibenzylamine as the main products.

**Figure 5:**
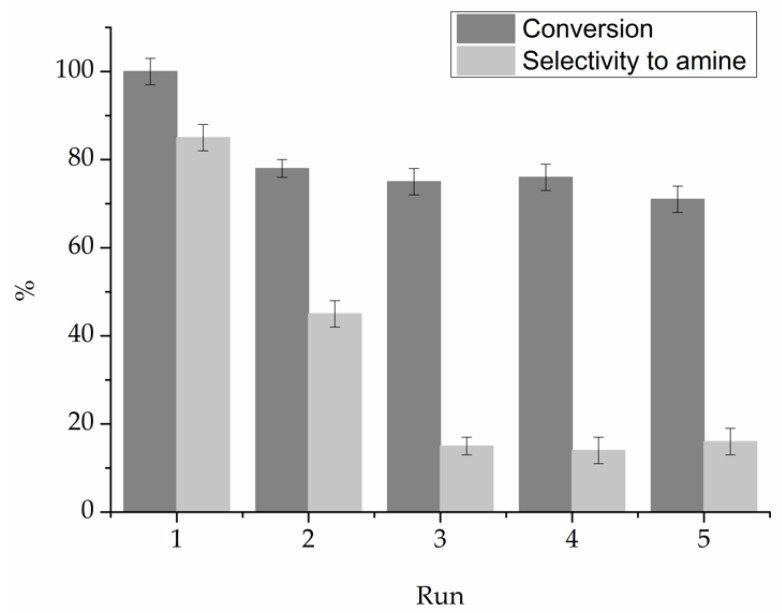
Benzyl alcohol conversion and selectivity to *N*-isopropylbenzylamine on recycling (6 h) over Au_SI_/NiO.

As the 1% Au_SI_/NiO catalyst showed the highest catalytic performance among the tested catalysts, stability tests were performed in order to investigate its potential reusability. The recycling consisted of filtering and reusing the catalyst for the next run without any further purification. After the first run, 1% Au_SI_/NiO showed a minor decrease in both the benzyl alcohol conversion and the yield to benzenemethanamine *N*-(1-methylethyl). Nevertheless, the activity remained stable for the successive three repetitions of the stability tests, but the catalyst significantly lost the capacity to hydrogenate the imine (Figure 5).

To understand the catalyst’s deactivation, the Au_SI_/NiO after reaction was characterized by XRD and TEM. XRD patterns did not show obvious modifications in the structure of the NiO support (Figure 1). A representative HAADF-STEM image of the catalyst after reaction shows a marginal change in the gold particle size (Figure 2c). Comparing the Gaussian fit, calculated from PSD, between the fresh catalyst (red) and after reaction (blue), the latter presents a slightly wider distribution with an increased average particle size of 5.3 nm (Table 1, Figure 2e). However, 1% Au_SI_/NiO is more effective in maintaining a narrow PSD compared to 0.7% Au_DP_/NiO (Figure 2f). Therefore, we believe preparing gold catalysts on NiO by SI synthesis leads to highly dispersed systems with stronger metal support interaction, which results in enhanced protection against particle agglomeration and/or detachment during reactions. The slight increase of the average Au particle dimension can be attributed to the partial removal of the protective agent during the reaction.

Nevertheless, it is not possible to validate this argument, due to the inability to distinguish between polyvinyl alcohol and other adsorbed products. As a result, the residual protective agent on the catalyst after reaction cannot be identified. However, the comparison of fresh and used catalyst after reaction did not allow us to completely understand the loss of the capacity of the catalyst to hydrogenate the intermediate imine. We extended the study to the amination of benzyl alcohol with different primary amines using Au_SI_/NiO to show the general applicability of our catalytic system, (Table 3). The catalyst fully converted benzyl alcohol and aniline with a good selectivity to the secondary amine (96%) at full conversion. The catalyst showed good activity in presence of linear and cyclic aliphatic amines, which are less reactive. For linear primary amines, a direct relation between length of the chain and reactivity was obtained. The order of activity and selectivity was the following: butylamine (52%) > hexylamine (36%) > octylamine (27%) in terms of activity, and butylamine (77%) > hexylamine (74%) > octylamine (22%) in terms of selectivity (Table 3). In Table 4, we present a comparison of Au_Si_/NiO with other Au systems reported in the literature for the reductive amination of benzyl alcohol with aniline, confirming the good performance of our catalyst.

## 3. Materials and Methods

### 3.1. Preparation of NiO

NiO was prepared according to the method of Villa et al. [23], with some parameter modifications. Ni(NO_3_)_2_·6H_2_O (26.8 mmol) (Sigma-Aldrich, purity >99.9%) and urea (Sigma-Aldrich, Milan, Italy, purity >99.5%) (urea/Ni 12:1 mol/mol) were added to 200 mL of water with magnetic stirring for 4 h at 80 °C. The Ni(OH)_2_ was separated from the solution by filtration and washed several times. The powder was dried at 100 °C in air for 2 h, and then calcined at 300 °C in air for 1 h.

### 3.2. Synthesis of Au Catalyst

Sol immobilization (Au_SI_): solid NaAuCl_4_·2H_2_O (0.051 mmol) and 1 mL of a PVA solution (1 wt %) were added to 200 mL of H_2_O (Au/PVA 1/0.5 wt/wt). After 3 min, a 0.1 M of NaBH_4_ solution (Au/NaBH_4_ 1/4 mol/mol) was added to the yellow solution under vigorous magnetic stirring. A ruby red Au(0) sol was immediately formed. Within a few minutes of their generation, the colloids (acidified at pH 2, by sulfuric acid) were immobilized by adding the support to the vigorously stirring solution. The amount of the support was controlled, in order to obtain a final Au loading of 1 wt % (on the basis of quantitative loading of the metal on the support). The catalysts were filtered and washed several times, and dried at 100 °C for 2 h.

Deposition precipitation (Au_DP_): the support (1 g) was dispersed in distilled water (approximately 10 mL/g of support) to which ammonia was added, to raise the pH to a value around 9. Solid NaAuCl_4_·2H_2_O (0.051 mmol, as having 1 wt %) was added to the support under vigorous stirring. The catalyst was filtered, and washed several times with water. The material was then suspended in distilled water, and a 0.1 M solution of NaBH_4_ was added (Au/NaBH_4_: 1/4 mol/mol) with vigorous stirring at room temperature. The sample was filtered, washed, and dried at 80 °C for 4 h.

Benzyl alcohol amination: the reactions have been performed using a stainless steel Parr reactor (50 mL capacity), equipped with heater, mechanical stirrer, gas supply system, and thermometer. A mixture of benzyl alcohol (7.2 mmol) and amine (7.2 mmol) was brought to a volume of 30 mL in xylene. The Au catalyst (BA/Au = 250 mol %) was added to the system, which was then stirred under 5 atm N_2_ at 150 °C. When the reaction was completed, the system was cooled to room temperature, and the mixture was filtered. Samples were removed periodically (0.2 mL) and analyzed using a HP 7820A gas chromatograph equipped with a capillary column HP-5 30 m × 0.32 mm, 0.25 µm Film, by Agilent Technologies, Italy. Identification of products was performed using a Thermo Scientific Trace ISQ QD Single Quadrupole GC-MS equipped with a capillary column HP-5 30 m × 0.32 mm, 0.25 µm Film, by Agilent Technologies. Authentic samples were also analyzed to determine separation times. Quantitative analyses with external standard method (*n*-octanol) was used. Recycling tests were carried out under the same experimental conditions. The catalyst was recycled in the subsequent run after filtration, without any further treatment.

Characterization: samples for transmission electron microscopy (TEM) studies were prepared by depositing small amounts of dry catalyst powder onto holey carbon copper grids. Micrographs and analytical studies by energy-dispersive X-ray spectroscopy (XEDS) analyses were performed in high annular dark field mode using a FEI Titan3 80–300 microscope operated at 200 kV. Digital Micrograph, TIA, and INCA software were used for the analysis of the TEM micrographs and XEDS spectra. The PSD, Gaussian fit, average particle diameter, and metal dispersion were calculated assuming a truncated cuboctahedron particle shape using Gauss software.

Metal content was verified by atomic absorption spectroscopy (AAS) using a Perkin Elmer 3100.

X-ray diffraction (XRD) experiments were performed on a Rigaku D III-MAX horizontal-scan powder diffractometer with Cu–Kα radiation, equipped with a graphite monochromator on the diffracted beam. The crystallite size was estimated from peak half width by using the Scherrer equation, with corrections for instrumental line broadening (β = 0.9).

## 4. Conclusions

We have demonstrated the enhanced activity of Au/NiO nanohybrids compared to Au/TiO_2_ systems in the reductive amination of benzyl alcohol. The resulting catalyst was more active in both dehydrogenation and reduction steps when compared to supported Au nanoparticles on TiO_2_. Au/NiO catalyst prepared by sol immobilization showed a better activity and stability compared to the ones prepared by deposition precipitation. In particular, the presence of a protective agent seems to be essential to stabilize Au nanoparticles during reaction when supported on nanostructured NiO. However, further studies are needed to understand the role of the protective agent during the reaction and to enhance the stability of the catalyst.

## Figures and Tables

**Table 1:** Statistical median and standard deviation of Au particles size.

Catalyst	Statistical Median (nm)	Standard Deviation σ
1% Au_DP/_TiO_2_	3.8 ^(a)^	1.1
1% Au_SI/_TiO_2_	3.5 ^(a)^	0.7
0.7% Au_DP_/NiO	3.4	2.0
0.7% Au_DP_/NiO after reaction	7.3	4.5
1% Au_SI_/NiO	4.8	2.2
1% Au_SI_/NiO after reaction	5.3	2.3

^(a)^ taken from reference [25].

**Table 2:** Activity and selectivity of Au_DP_ and Au_SI_ based catalysts for the reductive amination of benzyl alcohol with isopropylamine.

Catalysts ^(a)^	Conversion % ^(b)^	Selectivity (%)				
		*N*-isopropylbenzylimine	*N*-isopropylbenzylamine	Diisopropylamine	1	2
NiO	29	95	-	-	-	5
TiO_2_	<1	-	-	-	-	-
1% Au_DP_/TiO_2_	38	18	71	9	1	1
1% Au_SI_/TiO_2_	32	55	33	5	-	-
0.7% Au_DP_/NiO	58	17	83	-	-	-
1% Au_SI_/NiO	>99	1	85	-	1	11

^(a)^ Reaction conditions: isopropylamine (7 mmol), benzyl alcohol (7 mmol), total volume in xylene (30 mL), BA/Au: 250 mol %, 150 °C, 5 atm. N_2_, 1200 rpm; ^(b)^ conversion based on benzyl alcohol consumption, reaction time 24 h.

**Table 3:** Activity and selectivity of 1% Au_SI_/NiO for the reductive amination of benzyl alcohol with different amines.

Amine ^(a)^	pK ^(b)^	Conversion % ^(b)^	Selectivity		
			Imine	Secondary Amine	Others
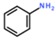	9.40	>99	2	96	2
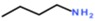	3.40	52	8	77	15
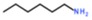	3.44	37	12	74	13
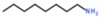	3.35	26	47	22	31
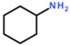	3.30	28	36	10	54

^(a)^ Reaction conditions: amine (7 mmol), benzyl alcohol (7 mmol), total volume in xylene (30 mL), BA/Au: 250 mol %, 150 °C, 5 atm. N_2_, 1200 rpm; ^(b)^ conversion based on benzyl alcohol consumption, reaction time 24 h.

**Table 4:** Activity and selectivity of different Au systems for the reductive amination of benzyl alcohol with aniline.

Catalyst	Time of Reaction (h)	Conversion %	Temperature (°C)	Metal/Substrate Ratio	Selectivity to Secondary Amine	Reference
Au_SI_/NiO	24	>99	150	1/250	96	This work
Au/ZrO_2_	22	82	110	1/65	60	[18]
Au/WO_3_	22	34	110	1/65	56	[18]
Au/ZrO_2_	22	99	110	1/25	94	[18]
Au/Al_2_O_3_	14	49	120	1/200	36	[22]
Au/MgO	14	36	120	1/200	16	[22]
Au/TiO_2_-VS	19	99	120	1/200	91	[22]
